# Remembering New Words: Integrating Early Memory Development into Word Learning

**DOI:** 10.3389/fpsyg.2013.00151

**Published:** 2013-04-01

**Authors:** Erica H. Wojcik

**Affiliations:** ^1^Department of Psychology, University of Wisconsin-MadisonMadison, WI, USA

**Keywords:** memory development, memory encoding, memory consolidation, memory retrieval, word learning, memory and learning, language development

## Abstract

In order to successfully acquire a new word, young children must learn the correct associations between labels and their referents. For decades, word-learning researchers have explored how young children are able to form these associations. However, in addition to learning label-referent mappings, children must also remember them. Despite the importance of memory processes in forming a stable lexicon, there has been little integration of early memory research into the study of early word learning. After discussing what we know about how young children remember words over time, this paper reviews the infant memory development literature as it relates to early word learning, focusing on changes in retention duration, encoding, consolidation, and retrieval across the first 2 years of life. A third section applies this review to word learning and presents future directions, arguing that the integration of memory processes into the study of word learning will provide researchers with novel, useful insights into how young children acquire new words.

## Introduction

What do children have to do to learn a new word? First, they must attend to and encode information about the referent, the label, and the association between the two. In other words, they have to learn about how the sounds in their language map onto objects, actions, and other properties of the world. However, in addition to learning this information, children also have to remember what they have encoded. Figuring out what the word “ball” refers to in one particular situation is helpful, but retaining this knowledge for future use is equally, if not more, important for word learning to be successful.

While we typically describe the acquisition of words as an inductive learning process, it is clear that it is also a memory process; children not only have to learn words, but they also have to remember them. However, most word learning studies do not investigate memory. A typical study consists of either (a) presenting an array of objects and testing how children disambiguate which referent matches a novel label or (b) teaching children several novel words and then testing what they have learned immediately after training. Because follow-up tests are rarely done (barring a few notable examples, such as Carey and Bartlett, 1978), what we know about word learning is largely limited to how children disambiguate and encode new words after a brief exposure.

Remembering words, though, is just as important as learning them; young children must be able to recognize and recall words in order to communicate, and they must be able to retrieve previously encoded words in order to update those representations with new information. Because of the importance of retaining novel words, memory processes need to be incorporated into word learning research. By reviewing what we know about early memory development and presenting several examples of how this literature is applicable to word learning research, this paper provides a new perspective that can be used to better understand the full process of how young children learn, and retain, new words.

## Memory Processes and Development

Psychologists have long divided adult memory into three constituent stages: encoding, consolidation, and retrieval (see Anderson et al., 1998). Encoding refers to the perception and first registration of a memory. Neurologically, encoding involves the primary sensory cortices and association cortices, as well as the hippocampus (see Eichenbaum and Cohen, 2001). Although word-learning researchers do not often use the term “encoding,” most experiments examine just that. For example, research has shown that infants encode perceptual information about object categories (e.g., Younger and Cohen, 1986). We also know that infants encode information about word forms, using prosody, known words, and transitional probabilities to segment words from a stream of speech (Jusczyk and Aslin, 1995; Saffran et al., 1996; Nazzi et al., 1998). Lastly, we know that young word learners can use cross-situational statistics, mutual exclusivity, syntactic bootstrapping, social cues, and other strategies and biases to encode the association between referents and labels (e.g., Naigles, 1990; Tomasello, 2000; Smith and Yu, 2008; Bion et al., 2012). Because these studies follow the widely used format of presenting infants with novel stimuli and immediately testing what they learned, they reveal what children are encoding about referents, sounds, and the relationships between them.

While encoding is an important aspect of memory, there are other processes involved in forming a representation that is remembered over time. After encoding, a perceptual trace is translated into a cortical memory trace that can be maintained over a longer period of time. This process is called consolidation (see Zola and Squire, 2000). Neurologically, consolidation is the re-encoding of a memory trace from the hippocampal formation to the cortex (Dudai, 2004). As memories consolidate, they become less susceptible to forgetting (see Wixted, 2004 for a review). While the role of consolidation and the mechanisms involved are still under debate, it is clear from both neurological and psychological data that consolidation is an important stage in long-term retention, with the key idea being that in order for memories to be retained, they must be successfully consolidated into cortex. If there is interference to this process, then it is likely that the memory will be forgotten.

The last stage of memory is retrieval, or the reactivation of a memory trace (see Buckner and Wheeler, 2001 for a review). When a memory is retrieved (after consolidation), the relevant cortical areas for that memory are activated (i.e., the visual cortex for visual components of memory etc.; see Wheeler et al., 2000). Retrieval has been shown to strengthen memory traces (Roediger and Karpicke, 2006), and thus the retrieval process is involved not just in recalling a memory, but also in successful continued retention.

Despite the importance of all of these stages for successful memory retention, most word learning studies either test children’s disambiguation of a novel word’s meaning (e.g., Halberda, 2003) or test word knowledge at one time point immediately after training (e.g., Smith and Yu, 2008). Thus, much of what we know about how children learn the meanings of words relates to on-line comprehension strategies or the encoding process. Interestingly, we know that young infants are able to both encode and retain lexical word forms (see Jusczyk and Hohne, 1997; Houston and Jusczyk, 2003; Swingley, 2005). For example, 7.5-month-old infants can remember a spoken word form for at least 24 h (Houston and Jusczyk, 2003). Less is known, though, about how infants retain representations of word *meanings*. Because young children must retain these representations in a stable semantic system, it is necessary to understand how novel word meanings are retained beyond initial encoding^1^.

The remainder of the paper will begin with a review of word learning studies that have investigated retention. Then, the infant memory literature will be reviewed, followed by an application of this review to the word learning literature. The research and ideas presented will be grounded in a developmental perspective, such that the focus will be on the characteristics of, and changes in, memory and word learning processes during the first 2 years of life.

## What We Know about Retention and Word Learning

A handful of studies have examined what infants remember about newly learned words over time. One of the first studies to examine the long-term retention of novel words was performed by Carey and Bartlett (1978). In this study, 3-year-olds were naturalistically introduced to a new word with the phrases, “Bring me the *chromium* one. Not the red one, the *chromium* one.” After 7–10 days, children were tested on their knowledge of this word with a range of tasks. It was found that after this delay, eight of the 19 children showed comprehension of the word in a forced choice task with nine total objects. Then, there was another delay of 10 weeks, after which the children were given two more exposures to the novel word, and then tested another 7–10 days later. Ten of the 19 subjects showed comprehension during this second testing cycle. This experiment was the first to demonstrate that children can learn and retain the meaning of a word after only a brief exposure.

Carey and Bartlett’s groundbreaking study inspired multiple generations of research examining how young children learn words. Interestingly, much of this work does not use a delay between training and test, and instead focuses on young children’s impressive ability to infer the meaning of a word when hearing it for the first time (for reviews, see Markman, 1990; Horst and Samuelson, 2008). While this ability to “fast map” a novel word was one aspect of Carey and Bartlett’s experimental design, they also tested whether the children could retain the newly learned word over a delay period. In the real world, children must infer the referents of novel words, but they also must retrieve words days or weeks after they hear them. Testing comprehension immediately after a teaching moment only tells you about what has been encoded. It does not tell you whether those children will remember that word the next time they encounter it.

There have been a handful of studies since Carey and Bartlett’s that have included a delay between training and test. For example, Goodman et al. (1998) taught 2-year-olds novel words using semantically informative sentences and then tested comprehension after a 24-h delay. They found that children could still understand the words after the delay period, demonstrating that 2-year-olds are able to use semantic context to learn and retain new words. Similarly, Woodward et al. (1994) found that both 13- and 18-month olds month olds show comprehension of a novel word that is directly labeled (i.e., “This is a dax!”) after a 24-h delay. Other studies have used similar designs to demonstrate that by one to one and a half years of age, children can retain a newly learned word for at least a day (Baldwin and Markman, 1989; Mervis and Bertrand, 1994; Markson and Bloom, 1997; Waxman and Booth, 2000; Jaswal and Markman, 2003; Spiegel and Halberda, 2011; Munro et al., 2012; see Horst and Samuelson, 2008 for a review).

Notably, though, the comprehension tasks used in these studies were relatively easy. In most cases, children had to pick out the correct referent from an array of objects that included the newly learned referent and several familiar referents. Recent studies have demonstrated that the saliency of novel objects significantly influences young children’s choices in comprehension tasks (Mather and Plunkett, 2012). When novelty is controlled for (by including foils that are equally as novel as the trained word), 2-year-olds do not show comprehension of newly mapped word after a 5-min delay in a pointing task (Horst and Samuelson, 2008; see also Kucker and Samuelson, 2011).

Additionally, the memory of a newly learned word continues to decay as time goes on. Vlach and Sandhofer (2012) tested 3-year-olds’ comprehension of an explicitly labeled novel word both 1 week and 1 month after training (controlling for novelty at test) and found that memory performance declined in a curvilinear manner over time. While about 70% of the participants showed comprehension of a novel word immediately after testing, this declined to just over 30% 1 week later, and to just over 10% 1 month later. This pattern demonstrates that while young children may show successful encoding of a word when they are tested immediately after training, their memory for that word drastically decays over time.

The studies mentioned above were designed to test *if* children can retain words across a delay. However, retention delays can also be used to test the strength of different types of novel word representations. If we assume that children forget words because their early representations are weak and decay over time, then stronger representations may survive longer delays. Along this line, Booth (2009) taught 3-year-olds six novel words. For half of the words, the children were given information about the causal properties of the referents, and for the other half they were given non-causal information. When they were tested minutes after training, there was no difference in comprehension between the two training conditions. After a delay of 6–15 days, though, a difference emerged: children only showed comprehension of the causally described words.

Two of the studies mentioned above also examined how encoding conditions affect retention. Horst and Samuelson (2008) showed that 2-year-olds *could* retain newly learned words for 5 min if the words were directly labeled instead inferred. Vlach and Sandhofer (2012) investigated whether the addition of supporting cues at training would increase 3-year-olds’ long-term retention of novel words. For this experiment, at training, the experimenter made the novel object more salient (by shaking it), repeated the label multiple times, and had the children produce the label. With these supports at training, 3-year-olds significantly improved their long-term retention, with just over 60% showing retention at the longest delay of 1 month (up from around 10% when none of the learning supports were provided). These studies indicate that the conditions surrounding the encoding of a novel word affect how long that word will be remembered.

The studies on retention thus far present a mixed picture of how well children 3 years of age and younger can retain words over time. On one hand, there is evidence that under the right circumstances, children can remember a word weeks or months after they hear it for the first time (e.g., Goodman et al., 1998; Booth, 2009; Vlach and Sandhofer, 2012). On the other hand, if children are not given semantic or linguistic support during training (and if the testing environment does not bias participants toward choosing the correct referent), children are oftentimes not successful at remembering novel words, even for a small amount of time (e.g., Horst and Samuelson, 2008; Bion et al., 2012; Vlach and Sandhofer, 2012). Thus, many questions remain surrounding the retention of newly learned words. For example, how does novel word retention change across development? Beyond encoding conditions, what else can account for successful retention? How do consolidation and retrieval processes affect word retention in young children? And, can these memory processes help explain developmental patterns that we seen in early word learning? Luckily, there is a vast amount of research on infant memory that can inform our understanding of the characteristics of novel word retention and word learning more broadly.

## Investigating Early Memory

Despite the fact that adult memory has been studied for over a century, a rigorous investigation of infant memory did not begin until the mid-1970s (Rovee and Fagen, 1976). A key reason for this late start stems from the fact that the majority of memory tasks used on adults involve verbal, and sometimes text-based, tasks. For example, one of the most influential paradigms in adult memory research involves providing participants with a list of words to memorize. The content of the list, the activities before and after training, the delay between training and test, and the conditions of the retrieval task can be manipulated to explore how adults encode, consolidate, and retrieve the list items (see Anderson et al., 1998; Wixted, 2004 for reviews). Because the tradition in memory research is to use verbal stimuli, it has been difficult to investigate memory in young, pre-verbal children.

There are many tasks that have been used to study the memory capabilities of children 4 years of age or older. This is due to the fact that once children reach this age, their language abilities are good enough that researchers can use explicit tasks to examine the properties of their memory. However, this increased language ability also makes the literature less relevant for word learning researchers; by the time children are 4 years old, they already know hundreds of words. In order to review the memory literature that is relevant for early word learning, then, we need to look to investigations of children under the age of three. Because this age group has very primitive language skills at best, most memory tasks that are used with adults and older children are not applicable. Fortunately, there are a few tasks that have been successfully used to test the memory of pre-verbal infants, two of which are operant conditioning and deferred imitation. Because these two tasks will be the primary focus of this review, they will be explained in depth.

### Operant conditioning

The infant operant conditioning paradigm was developed in the mid-1970s (Rovee and Fagen, 1976). Because the task requires a motor response, not a verbal one, it can be used to test infants as young as 2 months of age (Greco et al., 1986). In this paradigm, sometimes referred to as the mobile-kicking task, infants are placed in a crib, and one foot is tied with a ribbon to a mobile hanging overhead such that when the infant kicks, the mobile moves. Kicking is positively reinforced by the mobile movement, which conditions the infant to kick faster. It takes time for infants to learn to associate kicking with the movement of the mobile, and thus memory for the mobile can be assessed by testing whether infants still kick at an increased rate after a delay of varying lengths. Because the positive reinforcement is associated with a particular mobile, the visual characteristics of the mobile can be manipulated in the same way that word lists can be manipulated to test memory content.

There has been some debate surrounding what type of memory this methodology investigates, and in particular whether it taps into the implicit or explicit memory system (see Nelson, 1995; Rovee-Collier and Cuevas, 2009). As mentioned previously, though, this debate is beyond the scope of this paper, and the classification of the operant conditioning paradigm in terms of these two systems is not paramount to this review. Instead, what is important is whether experiments that employ this method can be informative to word learning researchers. Because the operant conditioning paradigm has been used to rigorously test various components of long-term memory in children under 2 years of age (see Rovee-Collier et al., 2001), the body of work can be helpful in shedding light on how memory development might influence early word learning.

### Deferred imitation

Another paradigm that has been widely used to study infant memory is deferred imitation. This paradigm was first used to examine infant memory in the mid-1980s (Meltzoff, 1985, 1988). Designed to test explicit memory in pre-verbal infants, the paradigm uses the imitation of a previously observed event as an index for how well the event was remembered. More specifically, infants first observe an adult experimenter model a sequence of events with an object or set of objects. They typically see around six different such events. After a delay of a day or more, infants are then given the objects (one set at a time), and the number of imitations of the experimenter’s previous actions is coded. Their performance is compared between subjects to another group of infants who never saw the actions modeled (i.e., Meltzoff, 1988), or within subjects to their own performance on a separate set of objects for which actions have not been modeled (i.e., Bauer, 2005). The conditions around the encoding of the actions, the delay between observation and imitation, and other parameters can be manipulated to test various aspects of infants’ memory for observed events. As with operant conditioning, deferred imitation tasks can be used with young infants, starting at around 6 months (Barr et al., 1996). Thus, findings from this task can be compared to those from the operant conditioning paradigm to make more generalized, less task-specific claims about infant memory.

Despite the difficulties in studying memory in pre-verbal infants, research with the operant conditioning and deferred imitation paradigms have increased our understanding of early memory development. The remainder of the paper will review what these methods have revealed about retention duration, encoding, memory consolidation, and memory retrieval in infancy. Following this review, a final section will integrate this review into what we know about word learning and propose some future directions.

## How Long Can Infants Retain a Memory?

One of the most basic questions in the memory development literature is, how long can infants retain a memory? Or, in other words, what is the rate of forgetting in infancy? This question is particularly relevant for word learning because of the assumption that children accumulate knowledge about a word’s meaning over multiple exposures (e.g., Smith and Yu, 2008; Nicol Medina et al., 2011). Many proposed mechanisms of word learning, including cross-situational statistical learning (Smith and Yu, 2008; Suanda and Namy, 2012) and Bayesian inference (Xu and Tenenbaum, 2007; Nicol Medina et al., 2011) involve an additive process in which children integrate multiple experiences with a word in order to form a representation. Because this assumption is a part of many theories, we need to investigate how long of a delay an infant can withstand before a memory can no longer be retrieved. Even more fundamentally, though, children need to remember a word in order to use it later (in either comprehension or production), and thus the forgetting rate is a major factor in successful word learning. While one word learning study has examined retention at multiple time intervals (Vlach and Sandhofer, 2012), this study only examined retention at 1 week and 1 month after learning, and only examined 3-year-olds compared to adults. There has yet to be a systematic investigation of how long young learners remember a novel word and how this changes during the first few years of life.

**Figure 1:**
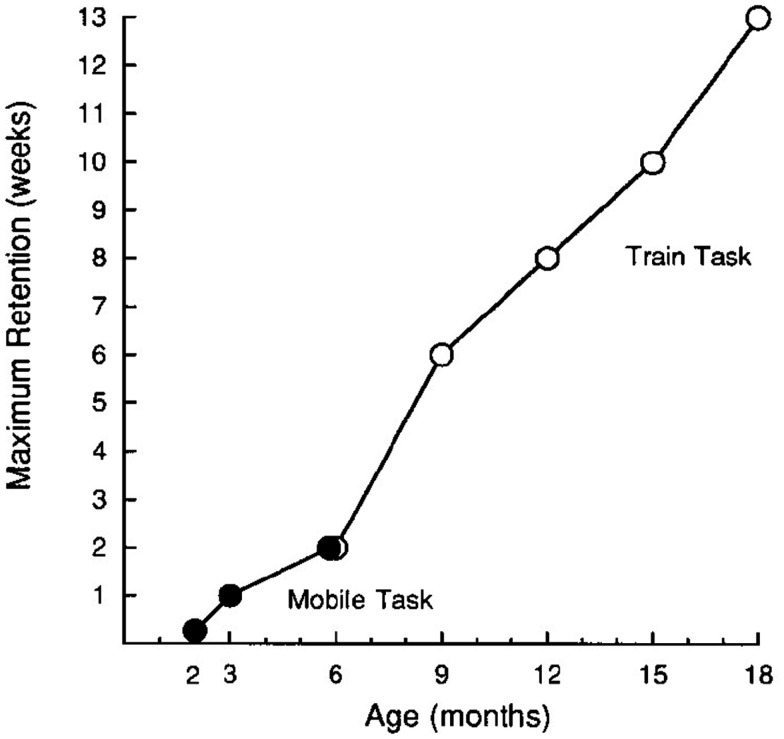
**Changes in maximum retention delay across development as demonstrated by the operant conditioning paradigm**. Adapted from Hartshorn et al. (1998).

Rovee-Collier and colleagues have investigated infants’ memory retention from 2 to 18 months using the operant reinforcement paradigm (Hartshorn et al., 1998). They demonstrated that 2-month-olds show recognition of the mobile after a 1-day delay period, but not longer (Vander Linde et al., 1985). As infants get older, this maximum duration of retention increases monotonically (see Figure 1). Three-month-olds recognize the mobile after a 1-week delay (Greco et al., 1990) and 6-month-olds recognize the mobile after a 2-week delay (Hill et al., 1988). For older infants, between 6 and 18 months of age, a variation of the mobile task is used. In this variation, infants are similarly trained with an operant condition task, but instead of kicking to move a mobile, they push a button to move a train around a track. This task also reveals a 2-week maximum retention delay for 6-month-olds (Hartshorn and Rovee-Collier, 1997). Nine-month-olds recognize the train after a delay of 6 weeks; 12-month-olds after 8 weeks; 15-month-old after 10 weeks; and 18-month-olds after 13 weeks (Hartshorn et al., 1998).

A similar pattern is seen with the deferred imitation task. Although the maximum retention delays are shorter (in designs with one exposure session, 6- and 9-month-olds show retention after a 24-h delay maximum, and 18-month-olds show retention after 2 weeks maximum; see Jones and Herbert, 2006 for a review) there is an analogous continuous improvement in retention in the first 2 years of life. This demonstrates that the memory increase seen in the operant conditioning paradigm is not specific to the task^2^. Thus, there is robust evidence that over the first year and a half of life, infants’ long-term memory continuously improves.

There are many factors that likely contribute to the steady improvement in memory retention in the first 2 years of life. One factor is encoding – if young children do not successfully encode a novel word, they will not be able to remember it later. Much of the word learning literature focuses on this one aspect of memory; we know that infants can use perceptual, social, and pragmatic information to encode new words. However, the memory literature has approached encoding from a different perspective that can be informative to word learning researchers.

## Encoding

As stated above, encoding refers to the first registration of a memory trace. In the early memory literature, tests of new memory representations after a delay of up to 10–15 min are purported to be assessing what has been encoded (e.g., Bauer et al., 2011). This is because consolidation takes several hours or longer in adult humans (McGaugh, 2000), and thus it is thought that memory tests after short delays tap into relatively unconsolidated memory traces (i.e., Davis et al., 2009). Notably, many word-learning experiments do not even test encoded representations, and instead focus simply on the disambiguation of the correct referent of a novel label. For example, Markman et al. (2003) tested 18- to 20-month olds’ use of mutual exclusivity to infer the meaning of a novel word. In one experiment, toddlers were presented with a familiar object and a bucket that held a novel object. The children were then asked, “Where’s the crimp?” The dependant measure was where the toddler reached or pointed after hearing the novel label. Because this frequently used paradigm only tests young children’s behavior upon first hearing the novel word, it does not address what kind of representation is encoded from the experience. While some word learning researchers do test novel word encoding, there is still much to understand about the encoding process in lexical acquisition.

One reliable finding in the infant memory literature is that with age, infants get faster at encoding. This point was first made in relation to infant habituation paradigms (Hunt, 1970; Hunter and Ames, 1988), in which infants are trained on visual or auditory stimuli, and then presented with familiar and novel test items. Infants tend to listen longer to familiar test items (termed a familiarity preference) if the stimuli are complex, and they tend to listen longer to novel items (a novelty preference) if the stimuli are simpler. The predominate explanation for this phenomenon is that once infants have successfully learned the training stimuli, familiar items are no longer interesting, and thus they attend more to novel stimuli. If they have not fully learned the information in the stimuli, they will attend more to familiar test items in order to continue learning (see Houston-Price and Nakai, 2004). Crucially, it has also been found that if older and younger infants are exposed to the same training stimuli, older infants do not need as long an exposure period in order to show a novelty preference. This has been taken as evidence that the older infants do not need as long to successfully learn, or encode, the stimuli.

Similar evidence on encoding time across development comes from the operant conditioning paradigm. While 2-month-olds need 3–6 min of exposure to the training mobile to learn the task (Greco et al., 1986), 3-month-olds only need 2–3 min (Greco et al., 1990), and 6-month-olds need 1 min (Hill et al., 1988). Likewise, in the deferred imitation paradigm, if younger children are given more exposure to the event sequences, they perform more like older children (they show more imitation at test; Barr et al., 1996).

What leads to this continuous increase in encoding speed? One possibility is that the development of brain systems related to attention in the first 2 years of life leads to faster encoding (see Colombo, 2001). More research is needed to understand how attention and encoding interact, and new advances in eye-tracking technology will lead to more work in this area (see Colombo, 2002). While we are still investigating why encoding speed increases across the first 2 years of life, it is clear that it does increase. Interestingly, it has also been found that across multiple ages, longer familiarization (i.e., longer encoding), leads to longer retention (Morgan and Hayne, 2006). Encoding is thus an important contributor to long-term memory retention, and the developmental changes in this process must have an effect on all processes that involve memory retention. Beyond encoding, another process that influences retention in infancy is consolidation.

## Consolidation

Consolidation is the post-encoding process in which memory traces are transferred, or re-encoded, from the medial-temporal system to the cortex (see McGaugh, 2000; Wixted, 2004). This process continues for weeks after a memory is encoded, and it results in a more stable and robust memory trace that can be retained over a long period of time (McGaugh, 2000). Studies that measure memory retention after a delay can be seen as measuring a combination of successful encoding and successful consolidation: both are needed for a memory trace to survive. While encoding differences have been studied in the context of word learning (e.g., Booth, 2009; Vlach and Sandhofer, 2012), researchers have not yet examined how consolidation affects whether retrieval is successful for young children.

In adults, successful incorporation of novel words into the lexicon depends on successful consolidation. Consistent with what is known about the neural bases of consolidation, the retrieval of newly encoded words leads to activation of medial-temporal areas, but after 1 day, retrieval activates cortical areas (Davis et al., 2009). Importantly, it is not until the words are consolidated into cortex that they interact with familiar words in a lexical decision task, indicating that this consolidation process is crucial for the integration of words into the semantic system. Similar results have been found for older children, aged 7–12 (Brown et al., 2012; Henderson et al., 2012).

Despite these findings, the role of consolidation in early word learning is still unclear. Young children, and particularly infants, do not necessarily learn via the same strategies and neural mechanisms as adults, and while consolidation has long been studied in adults (as well as in non-human animals and patient populations; McGaugh, 2000), only recently have researchers begun to examine consolidation in infancy (see Bauer, 2006; Bauer, 2007 for reviews). Brain areas in involved in adult consolidation – specifically the prefrontal cortex and dentate gyrus – continue to mature into early childhood (Huttenlocher and Dabholkar, 1997; Zola and Squire, 2000; Eichenbaum and Cohen, 2001; see Bauer, 2004 for a review). It has been hypothesized that because of the late development of these areas, the consolidation process is a crucial aspect of memory development to study (Bauer, 2006).

One of the difficulties in studying consolidation behaviorally is that in order to isolate this process, one has to ensure equal encoding across participants. The operant conditioning paradigm uses kicking rate immediately after training as a measure of encoding, and researchers have found that this kicking rate is statistically similar for not only the majority of infants within an age group, but also across age groups (e.g., Hartshorn et al., 1998). Thus, researchers have concluded that in this task, the changes in memory retention across development are due to consolidation, not encoding (see Rovee-Collier et al., 2001). However, no studies using the operant conditioning paradigm have explicitly controlled encoding levels, or matched participants on encoding in their analyses, and thus it is difficult to reach any definite conclusions about the role of consolidation in infant memory from operant conditioning studies. Studies using deferred imitation, though, have more systematically investigated consolidation across early development.

There is evidence that infants’ ability to successfully consolidate a memory significantly improves within the first year of life (Bauer, 2004, 2005). For example, Bauer (2005) presented 13-, 16-, and 20-month olds with a series of novel sequences. Crucially, after training, infants were given a chance to imitate the events. Their performance was used as a measure of encoding. After a 1-, 3-, or 6-month delay (between subjects), infants’ memory for all six sequences was tested. When infants were matched for level-of-encoding based on their initial imitation scores, there were remaining developmental differences, with older children outperforming younger children on the retention trials. Bauer concluded that because encoding was controlled for, the results indicate that changes in consolidation contribute to the differences in retention across development.

Another study used the deferred imitation task to examine how different encoding conditions lead to different levels of consolidation. In this study, 20-month-olds were taught 12 sequences via three trial conditions: watch, imitate, or learn-to-criterion (Bauer et al., 2011). For the “watch” trials, the infants simply observed the experimenter perform the sequences, and their encoding was measured 15 min later. For the “imitation” trials, infants were given a chance to imitate the actions immediately after they were presented. For the “learn-to-criterion” trials, infants were allowed to watch and imitate the sequences until they completed them correctly. The infants were then tested several days later (one to four) to measure retention.

To isolate the effect of consolidation, the researchers analyzed the retention scores of the infants who demonstrated complete encoding of sequences in all three trial conditions (as measured by their immediate imitation scores). They found that sequences that were “learned-to-criterion” were remembered better than those that were presented in watch or imitate trials, despite equal encoding. Moreover, sequences that were imitated were remembered better than those that were simply watched. These results demonstrate that while infants successfully encode memories under many conditions, not all learning conditions lead to successful retention.

In addition to behavioral studies, there is neurological evidence that consolidation accounts for unique variance in long-term retention in infancy. Bauer et al. (2003) combined EEG with a deferred imitation task to examine the contribution of both encoding and consolidation to 9-month-olds’ long-term retention. The participants’ long-term retention for six sequences was assessed via imitation scores after a 1-month delay. In addition to collecting the behavioral data, Bauer et al. recorded event-related potentials (ERPs) as the infants viewed both the trained (familiar) sequences and novel sequences. ERPs were recorded both immediately after training and 1 week later. The researchers were specifically interested in the middle-latency component (Nc), as this component is associated with recognition memory in infants (De Haan and Nelson, 1997). By comparing the characteristics of the Nc component for familiar and novel sequences at both time points, the researchers could determine the strength of the memory for the sequences both after encoding and after consolidation.

The behavioral data showed individual differences in retention after 1 month. Interestingly, while there was no relationship between retention scores and the ERP signatures recorded immediately after training, there was a relationship between retention scores and ERPs recorded 1 week after training. Specifically, the infants who successfully imitated the sequences after 1 month showed significantly different latencies to peak Nc for novel and familiar sequences at the 1-week recording. Infants who did not recall the sequences showed no difference in ERP recordings between novel and familiar sequences. These results demonstrate that consolidation failure, not encoding failure, leads to unsuccessful retention; infants who did not retain the events did show successful encoding, but did not show successful consolidation.

Together, the studies by Bauer and colleagues demonstrate that the consolidation process that occurs between encoding and retrieval has an affect on retention in the first 2 years of life. More specifically, they demonstrate that the variability in retention across development can be partially explained by differences in consolidation. The cross-sectional study (Bauer, 2005), combined with the ERP results of Bauer et al. (2003) suggest that younger children may successfully encode a new memory, but may have difficulty consolidating the representation. Additionally, while infants can successfully encode memories with little support, they may fail to consolidate those memory traces (Bauer et al., 2011). This research shows that in infancy, the neurological processes that occur between encoding and retrieval affect whether a memory will be retained. There is also research showing that the activities that occur during this time period, specifically whether or not infants sleep, affect consolidation.

### Sleep and consolidation in infancy

Sleep is often required for optimal consolidation of memory in adults (i.e., Stickgold, 2005). A couple of recent studies have applied this phenomenon to infants and their ability to learn a new grammar. Both studies investigated 15-month-olds’ ability to learn an AxB non-adjacent dependency grammar. In this grammar, A, x, and B are all syllables wherein A always predicts B, but x can vary. This is a common pattern in real languages.

To examine the affect of sleep on learning, Gómez et al. (2006) familiarized infants with a novel AxB language by playing them strings of syllables that fit this pattern. There was then a 4-h delay during which half of the infants took a nap. To achieve these conditions, infants were familiarized with the grammar before their typical naptime or at a different time of day. After the delay, both groups of infants were tested on how well they learned the grammar using the Headturn Preference Procedure. The infants were tested both on whether they could recognize familiar AxB sequences, and also on whether they could generalize their knowledge to new sequences that fit the pattern. While both groups recognized the familiar strings, only the infants who napped during the delay period succeeded in generalizing the grammar to new stimuli. Another study used the same paradigm and age group, but instead of a 4-h delay, a 24-h delay was employed between training and test. Again, half the infants napped within 4 h of familiarization and half did not (Hupbach et al., 2009). In this study, only the infants that napped showed recognition of even the familiar strings a day later.

These studies show that napping affects both whether infants consolidate a memory as well as the quality of that memory. More specifically, sleep can lead to more successful retention and to a more generalized representation of a memory. While the process by which sleep aids consolidation is still being investigated, these studies show that sleep affects memory consolidation in infancy. Combined with the deferred imitation studies and the slow development of the medial-temporal system, this work provides increasing evidence that infants may fail to retain a memory not because they forget that memory over time, but because they fail to consolidate the memory into a more stable cortical representation.

One obstacle in the study of consolidation is that it is difficult to disentangle effects of consolidation and retrieval. In the studies reviewed above, consolidation is measured by having participants retrieve a memory at different time points to assess the strength of the representation. Therefore, in addition to assessing how well a memory is consolidated, these studies are also measuring infants’ retrieval of that memory. The interpretation of the reported studies is that task failure is a result of unsuccessful consolidation, but it could be that it is a result of unsuccessful retrieval; the infants could have difficulty re-activating the relevant representation. Researchers must keep this confound in mind when they explore consolidation in both infants and adults.

A related problem in some of the studies reviewed above stems from the fact that when consolidation is measured at an initial time point, infants must retrieve the representation to demonstrate recognition. Thus, when long-term retention is measured at a second time point, it is measuring not just the representation of the initial memory, but also the effect of the initial retrieval. A question arises from this confound: what is the affect of retrieval on long-term retention in infancy?

## Retrieval

In addition to encoding and consolidation, the retrieval process can also influence retention. For example, Anderson et al. (1994) demonstrated that past-retrieval influences memory retention in adults. In this study, adults first memorized a list of arbitrary word pairs. Half of the memorized pairs were then retrieved in a cued recall design: the first word in a pair (the cue) was presented, and the participants had to recall the second word. Afterward, the participants’ memory for all the word pairs was assessed. The researchers found that not only were the previously retrieved items recalled more accurately at test, but also the recall of the unpracticed items was impaired. This study and others (see Wixted, 2004) have demonstrated that when words are retrieved, they become more robust.

The effect of retrieval on memory retention in infancy has been examined with the operant conditioning paradigm. We know that the maximum duration of retention for a memory increases linearly over the first year of life (see previous discussion). Given this finding, researchers asked whether the lifespan of a reactivated memory shows the same developmental pattern. To address this question, researchers first trained infants in an operant conditioning paradigm (using the mobile task for infants under 6 months of age, and the train task for infants 6 months and older). This training was followed by a delay period that was a week longer than maximum duration of retention for that age group, as found in previous studies (e.g., Hartshorn et al., 1998). After this delay, the infants were re-exposed to the training mobile. Crucially, the participants were not re-trained; they were simply allowed to view the familiar mobile. The lifespan of the reactivated memory was studied by employing a variable length of delay (between subjects) before the final test.

Across multiple studies, it was found that for 3-, 6-, 9-, and 12-month-olds, the maximum duration of retention is the same for the reactivated memory as it is for the original memory (1, 2, 6, and 8 weeks, respectively; Rovee-Collier et al., 1980; Hildreth and Rovee-Collier, 2002; Hildreth et al., 2003). In other words, a reactivated memory is retained for as long as the original memory, even if only a cue to the original memory is presented. In terms of retrieval terminology, this study shows that when an infant retrieves a memory, they do not just reactive it for a brief period of time. The act of retrieving leads to a robust memory trace that is remembered for just as long as the original (similar results are found using the differed imitation paradigm; Barr et al., 2005).

The most dramatic increase in retention due to retrieval can be seen in Rovee-Collier et al. (1999), which examined the effect of reminders on the retention duration of 2 month olds. The infants were trained on the mobile-kicking task. After two training sessions (separated by 24 h), infants were reminded every 3 weeks with a 3-min presentation of the mobile to reactivate the infants’ memory. After six reminder sessions (21 weeks past training), infants still showed above baseline kicking at test. None of the control infants, who received no reminders, showed this effect. In other words, with periodic reminders, a 2-month-old infant can retain a memory for the same length of time as a 24-month-old. Retrieving a memory can affect how long that memory is retained, even for young infants.

## Applications to Word Learning Research

This review of the infant memory literature has demonstrated that there are changes in how long a memory is retained across the first year of life, and that retention is influenced by changes in the encoding, consolidation, and retrieval processes. By taking this literature and applying it to word learning, the following section provides several examples of how a memory perspective can increase our understanding of word learning and lead to novel insights.

### Retention duration and word learning

The increase in the maximum duration of retention across the first 2 years of life (see Figure 1) has interesting implications for the study of the word learning. Recent studies have shown that infants already have some words in their lexicon by 6 months of age (Bergelson and Swingley, 2012). However, it is not until about 18 months that word learning hits its stride (Goldfield and Reznick, 1990; Mills et al., 1997; although, see McMurray, 2007; Fazly et al., 2010 for alternative analyses of the rate of vocabulary development). The traditional explanation for this increase in the rate of vocabulary development, or vocabulary spurt, is that there is a qualitative change in the mechanisms that support word learning. For example, one explanation is that children shift from associating labels and referents to linking labels to categories of referents (Nazzi and Bertoncini, 2003). Another explanation is that the spurt is driven by the emergence of word learning constraints, such as mutual exclusivity (e.g., Markman, 1990). These qualitative shift hypotheses are domain-specific. That is, they suggest that the vocabulary spurt is due to changes to language learning in particular.

However, a more parsimonious explanation for the vocabulary spurt is that it is related to more domain-general changes. One domain-general analysis of the vocabulary spurt is that an increased rate of learning is the by-product of any learning problem in which items are learned in parallel and with varying levels of difficulty (McMurray, 2007). However, the fact that there is a continuous decrease in forgetting rate during the first 2 years of life could also have an effect on the rate of word learning across that period of time. Recall that a 6-month-old infant’s maximum duration of retention is one sixth as long as an 18-month-old’s. It is possible, therefore, that the increased rate of word learning is partially related to memory development (see Dapretto and Bjork, 2000). In fact, a recent study has shown that while 16- and 20-month-olds can both integrate information from multiple exposures to learn a novel word, only 20-month-olds can integrate information if those exposures happen further apart in time (Vlach and Johnson, in press). This result suggests that there are developmental differences in word learning that are due to changes in how long infants can retain a memory.

In spite of these findings, the study by Vlach and Johnson (in press), as well as other studies that have examined how infants and adults learn words across multiple situations (e.g., Smith and Yu, 2008; Mather and Plunkett, 2009; Smith et al., 2011; Suanda and Namy, 2012; Trueswell et al., 2013) use a very short interval between each exposure. The novel words are still presented within one experimental session. In order to demonstrate whether infants are indeed able to accumulate word knowledge over time, we need to study whether they can integrate exposures that are separated by hours, days, or even weeks. Because we know that 6-month-olds forget an observed event after 24 h (Barr et al., 1996), it is possible that early on in the word learning process, maximum retention duration is a limiting factor in learning across multiple exposures. Future studies that examine how long-term memory fits into cross-situational word learning are needed in order to demonstrate that this strategy is viable in the real world. Additionally, research on the interaction between word learning and memory development will help provide insight into the vocabulary spurt.

### Encoding and word learning

As infants get older, they not only retain memories for longer; they also need less time to encode a given memory (see previous discussion). The fact that younger infants need more exposure to stimuli to successfully encode the information has not been explored in the word learning literature. While researchers often adjust the length of the familiarization or training period for novel word studies in order to ensure learning, the theoretical significance of this adjustment is rarely, if ever, discussed. It would be interesting to look at the number of training trials required in novel word learning studies for different aged children. A systematic examination of the required amount of novel word training across development would reveal whether the continuous decrease in encoding time applies to word learning as well.

If younger children do need more time to successfully encode new words, it is likely that more generally, younger children require more support at any given word learning moment. We know that more explicit labeling of novel words can lead to better encoding for both 2 and 3 year olds (Horst and Samuelson, 2008; Vlach and Sandhofer, 2012). Thus, for younger children, novel word labeling moments may need to be more explicitly highlighted in order to be successful. Recent studies using head-mounted cameras have found that the most successful word learning moments for 18-month-olds are those in which the children are holding the novel object themselves while the parent says the label (see Yu and Smith, 2012). These moments are particularly important given encoding constraints because the children can control the amount of time they spend attending to, and thus encoding, the newly labeled object. If this is the case, as infants get older and need less time to encode new words, they may rely less on these self-controlled encoding moments for word learning.

While the fact that younger infants need more time and support to encode new representations has implications for word learning, understanding the mechanisms behind successful encoding could be even more useful. In particular, as mentioned previously, the role between attention and encoding needs to be further explored (Colombo, 2001, 2002). Interestingly, there is evidence that visual attention may not be related to the encoding of new words in a straightforward way; visual attention patterns of 12- and 14-month-olds during a cross-situational word learning task were not correlated with successful encoding (Smith and Yu, 2012). As memory researchers continue to investigate the role of attention and other lower level mechanisms in encoding, word learning researchers need to continue to explore how these mechanisms affect word learning across development.

### Consolidation and word learning

Research with the deferred imitation task has demonstrated that there are multiple factors that contribute to whether or not an infant’s memory is successfully consolidated, including age, encoding conditions, and sleep (Bauer, 2005; Hupbach et al., 2009; Bauer et al., 2011, respectively). This work can also help to shed light on the relationship between word learning and memory. For example, recall the word learning study in which 3-year-olds were taught either causal properties of novel words or other, non-causal properties (Booth, 2009). While there were no differences in how well the children encoded the novel words (they showed equal comprehension of the words immediately after learning), there were differences after a 6–15 days delay, such that infants were more likely to remember the novel words if they had been taught a causal property. Like Bauer et al. (2011), the findings suggests that the conditions surrounding encoding affect retention via consolidation. While the type of learning condition did not affect how well the participants encoded a word’s referent, it did affect how well the word was consolidated. Thus, it appears that consolidation effects can be found in word learning tasks (see also Horst and Samuelson, 2008; Vlach and Sandhofer, 2012), not just deferred imitation.

While word learning studies have begun to explore the effect of encoding conditions on consolidation, researchers have not examined how age or sleep affect the consolidation of newly learned words. Investigating how age affects novel word consolidation early in development is necessary given recent findings that children are able to disambiguate novel words earlier than they are able to encode those novel words (Horst and Samuelson, 2008; Bion et al., 2012). This finding is enlightening because it shows that due to encoding difficulties, the mutual exclusivity bias, which has traditionally been thought of as a crucial early word learning strategy (Halberda, 2003), may not be helpful until children are older.

Along these lines, it is possible that many word learning strategies that are currently being investigated lead to the successful encoding of a word, but do not result in successful consolidation, particularly for younger children. It may be that while 30-month-olds can use mutual exclusivity to encode a novel word association (Bion et al., 2012), they fail to consolidate that representation, and thus may not be able to comprehend the word days later. Older children, though, may be able to both encode and consolidate a novel word that is learned via mutual exclusivity. In order to incorporate memory into how we think about word learning, we need to examine how differences in consolidation across development affect the use of word-learning strategies to not only encode novel words, but also to consolidate those words into a stable lexicon.

The last factor that contributes to successful consolidation in infancy is sleep (Gómez et al., 2006; Hupbach et al., 2009). Interestingly, a crucial aspect of semantic memory in adulthood is the fact that semantic knowledge includes both specific, episodic details and a more abstract, generalizable concept that can be flexibly applied to new situations (McClelland et al., 1995). Just as grammar patterns (such as those studied by Gómez and colleagues) must be generalized, therefore, so do word representations. Gómez’s work demonstrates that consolidation during sleep can aid in this type of generalization. It would be interesting to study the effect of sleep on the quality of novel word representations. More broadly, one could also study individual differences in sleep and how these differences relate to vocabulary growth and word knowledge. There is evidence that sleep is necessary for novel word consolidation in older children (aged 7–12 years; Henderson et al., 2012), but because most areas of the medial-temporal system are already close to developmental maturity by this age, it is unclear whether these results are transferable to infants who are in the throes of word learning. By studying the affect of sleep on novel word retention and generalization in young children, researchers will better understand how consolidation affects early word learning.

Researchers are just beginning to understand the consolidation process in both adults and children. Because the consolidation of words from the medial-temporal system into the cortex is a crucial process for successful novel word retention in adults and older children (Davis et al., 2009; Henderson et al., 2012), developmental psychologists need to study the role of factors such as age and sleep on the consolidation of novel words in infancy. As our understanding of consolidation improves, early word learning researchers can incorporate findings into subsequent experiments.

### Cued retrieval and word learning

Lastly, the literature on how retrieval affects memory in infancy – specifically the fact that pre-verbal infants can use cues to retrieve, and thus reactivate, a memory (e.g., Hildreth and Rovee-Collier, 2002) – also has implications for word learning. Studies of word learning typically expose infants to novel words with one training session, and then test what they learned with one testing session. However, young children encounter words, or cues to those words, many times a day. Compared to other memories, words are retrieved very frequently. An infant may only need to activate the memory of her aunt every several months, but she hears the word “milk” multiple times a day. Lexical activation studies demonstrate that when 2-year-olds hear a word label, they activate (or retrieve) its semantic representation (Willits et al., under review; Wojcik and Saffran, in press). Since some words are frequently heard, and thus frequently retrieved, we need to understand how this reactivation affects the memory of a newly learned word.

Interestingly, a word’s frequency of use, particularly in child-directed speech, is correlated with the age at which that word is acquired (Goodman et al., 2008; Roy et al., 2009). While word frequency likely entails more encoding opportunities, it also means more opportunities for a lexical representation to be reactivated. Thus, it is likely that the retrieval of a word across time helps strengthen that memory so that it is more successfully retained. Studying novel word retrieval effects is particularly important in light of the fact that infants may not be successful at retaining a novel word after a brief exposure due to incomplete consolidation (see previous discussion). If infants need more support to successfully consolidate a word, then the effect of additional retrieval opportunities should be examined.

A first step in applying the retrieval literature to word learning would be to test how cued retrieval affects word retention. Recall Rovee-Collier and colleagues’ investigation of the effect of simply presenting a previously trained mobile on infants’ retention of the mobile-kicking association. Similarly, language researchers can test how experiencing cues to a previously learned novel word – such as viewing the referent or hearing the label – affect its long-term retention. In addition to increasing our understanding how children form a stable lexicon despite the fact that they show poor retention under many circumstances (as discussed previously), examining the effect of cued retrieval on novel word retention will help explain the relationship between word frequency and age of acquisition.

## Where Do We Go from Here?

There have been decades of research on memory processes and development during the first 2 years of life, particularly in the areas of retention duration, encoding, consolidation, and retrieval. The previous section demonstrates that there are many ways to apply this research to help push the study of word learning in new directions. More broadly, though, it is necessary for researchers to move beyond studying how infants first map new words onto referents and integrate memory processes into how we think about word learning.

Researchers have begun to study what Carey and Bartlett (1978) called “slow-mapping,” or the accumulation of word knowledge over the course of a long period of time (see Swingley, 2010). This has caused researchers to consider to how conceptual development and word learning interact (Carey, 2010), how multiple experiences can accumulate to form a more accurate word representation (Smith and Yu, 2008; Nicol Medina et al., 2011), and how different types of information can interact to form complex word knowledge (Graf Estes et al., 2007; Yurovsky et al., 2012). Despite this emerging literature, there is still very little research on how the prolonged process of learning words interacts with the developing memory system in infants and toddlers.

To address this gap, researchers need to first view the process of word learning from a more comprehensive perspective. While there are some leaning constraints that are specific to word learning (either because children have learned these strategies, i.e., Samuelson, 2002, or because they are innate, i.e., Markman, 1990), it is clear that word learning relies on perceptual and cognitive processes that are more domain-general. If we want to fully understand word learning, we need to think about how the characteristics of various cognitive systems across development affect this process. In terms of memory, this means that researchers need to work to integrate what we know about how infants remember and store events into their theories of early word learning.

Secondly, researchers must think about how the parameters of small laboratory tasks affect our theories. Because we must work within the attention span of infants, word-learning experiments often last about 10 min. This timeframe obstructs our ability to understand the role of time, and thus memory, in the learning process. By developing new paradigms to test how infants use knowledge over time, we can better understand how memory fits into word learning, and thus gain novel insights into the impressive ability of young children to so efficiently form a stable lexicon.

## Conclusion

Word learning has traditionally been studied as an isolated, domain-specific problem of inducing the correct referents for a given label. However, word learning is a much more complex problem that can be grounded in other cognitive processes. Yes, children must first map labels onto referents, but they must also encode, consolidate, and retain these representations. The process of how infants and young children encode, store, and retrieve representations has been studied rigorously for half a century, and yet this research has rarely been used to inform our study of word learning.

This review has demonstrated that study of early memory development can be used to inform our understanding of early word learning. However, it is also possible for early word learning research to contribute to what we know about memory development. It is challenging to study memory in pre-verbal children. Interestingly, though, despite the fact that we are still discovering how infants remember new words, we know that they *can* – infants show comprehension of words within their first year of life. Thus, further investigation into how infants can retain novel words over a long period of time will also help us understand early memory development.

## Conflict of Interest Statement

The authors declare that the research was conducted in the absence of any commercial or financial relationships that could be construed as a potential conflict of interest.

## Footnotes

^1^In addition to the encoding, consolidation, and retrieval phases, researchers have carved up memory in many other ways as well, including into multiple systems. One popular system division is between implicit and explicit memory (see Graf and Schacter, 1985). However, the utility and veracity of this distinction controversial (see, for example, Buchner and Wippich, 2000), and additionally, the dichotomy originates from the adult literature (see Rovee-Collier, 1997; Rovee-Collier and Cuevas, 2009). Because this review focuses on memory in infancy and its contribution to word learning, the implicit/explicit dichotomy is both beyond the scope of this paper and tangential to the thesis of the review. Rather than classifying experimental tasks as tapping into the implicit or explicit system, the paper will analyze the literature from the perspective of what the results tell us about the character of early memory as it relates to word learning.

^2^The shorter maximum delay seen in deferred imitation also demonstrates that retrieval demands affect memory tasks. Six-month-old infants may still have a memory of an event sequence past 24 h, but may not be able to activate a motor plan to imitate the actions. Variability in task demands can have implications for word learning, but that is beyond the scope of this paper.
